# Developmental and epileptic encephalopathies – therapeutic consequences of genetic testing

**DOI:** 10.1515/medgen-2022-2145

**Published:** 2022-09-22

**Authors:** Steffen Syrbe

**Affiliations:** Division of Paediatric Epileptology, Centre for Paediatrics and Adolescent Medicine, University Hospital Heidelberg, Im Neuenheimer Feld 430, 69120 Heidelberg, Germany

**Keywords:** developmental and epileptic encephalopathy, monogenic etiology, children, precision treatment

## Abstract

Developmental and epileptic encephalopathies comprise a heterogeneous group of monogenic neurodevelopmental disorders characterized by early-onset seizures, marked epileptic activity and abnormal neurocognitive development. The identification of an increasing number of underlying genetic alterations and their pathophysiological roles in cellular signaling drives the way toward novel precision therapies. The implementation of novel treatments that target the underlying mechanisms gives hope for disease modification that will improve not only the seizure burden but also the neurodevelopmental outcome of affected children. So far, beneficial effects are mostly reported in individual trials and small numbers of patients. There is a need for international collaborative studies to define the natural history and relevant outcome measures and to test novel pharmacological approaches.

Epileptic encephalopathies are early-onset severe epilepsy syndromes. An increasing number of genes are identified in about half of affected infants [1]. These distinct monogenic neurodevelopmental disorders (NDD) are now classified as developmental and epileptic encephalopathies (DEE), based on the genetic cause. The classification as monogenic DEEs acknowledged that in most cases the abnormal neurodevelopment is related to the genetic defect rather than seizure burden and abnormal electroencephalography (EEG) activity (Infobox 1). Characterization of functional genetic effects on cellular signaling defined potential novel targets for individualized treatments in these refractory epilepsies with a poor prognosis.

**The epileptic encephalopathies of childhood are electroclinical epilepsy syndromes with recognizable seizure types and EEG patterns (Infobox** **2****). Epileptic encephalopathies as electroclinical syndromes are classified by the International League Against Epilepsy (ILAE) [****2****], [****3****].** They are distinguished from isolated developmental disorders or developmental encephalopathies (NDD). In epileptic encephalopathies, the epilepsy and epileptic activity itself contribute to the development of the developmental disorder. In addition to intellectual disability (ID), associated neurological disorders, such as autism and movement disorders, often occur. The concept of an epileptic encephalopathy implied the hope that successful therapy of epilepsy and abnormal EEG could improve development, which, however, has not been demonstrated for all epileptic encephalopathies. When specific causes and a genetic predisposition are demonstrated, the prognosis for most childhood epileptic encephalopathies is poor.

In developmental encephalopathies, seizures may be absent or may occur only as part of the disorder. In many genetic disorders with epilepsy, a combination of developmental (e. g., genetic predisposition with a proven CDKL5 variant in a girl with atypical Rett syndrome) and epileptic encephalopathy (e. g., West syndrome with regression in infancy in the same patient) is present (see Infobox 1). The ILAE classifies epileptic encephalopathies according to age, seizure type, and EEG pattern (electroclinical syndrome). In this context, the main childhood epilepsy syndromes are defined phenotypically according to age [4] (see Infobox 2). Recently, the ILAE has proposed a novel classification for the epilepsy syndromes, which includes novel names and abbreviations for many of the age-related epileptic encephalopathies [2], [3].

**Infobox 1:** Frequently used terms and abbreviations in epileptic encephalopathies.

DEE	Developmental and epileptic encephalopathy	Genetic disorder often associated with a specific epileptic encephalopathy phenotype, e. g., KCNQ2 encephalopathy, which often begins as neonatal Ohtahara syndrome. The abbreviation is now used for all monogenic developmental disorders with epilepsy in the Online Mendelian Inheritance in Man (OMIM) database.
EE	Epileptic encephalopathy	Encephalopathy in which epileptic activity itself causes a more severe additional impairment of cognition and behavior that contributes to the developmental disorder beyond the underlying pathology (e. g., idiopathic West syndrome). Defined by the International League against Epilepsy as distinct electroclinical syndromes.
EIEE	Early infantile epileptic encephalopathy	Epileptological name for Ohtahara syndrome as a neonatal encephalopathy with tonic seizures and a suppression burst pattern. Genetic classification: Previous term, used in OMIM for various infantile developmental disorders with the leading symptom of epileptic seizures.
EME	Early myoclonic encephalopathy	Neonatal-onset early myoclonic encephalopathy with polymorphic epileptic and nonepileptic myoclonia, other seizure types, and a suppression burst pattern in the EEG.
EOEE	Early-onset epileptic encephalopathy	Not clearly defined term for epileptic encephalopathies with onset within the first 6–12 months. The abbreviation is often used as an umbrella term for neonatal epileptic encephalopathies with a suppression burst pattern.
IS	Infantile spasms	Abbreviation for the predominant seizure type in West syndrome (short tonic flexion). Also used as an alternative term for West syndrome (combination of infantile/epileptic spasms with hypsarrhythmia and associated developmental disorder).
NDD	Neurodevelopmental disorder	Developmental disorder or developmental encephalopathy from a distinct etiology (e. g., Rett syndrome with proven pathogenic variant in *MECP2*).

**Infobox 2:** Epileptic encephalopathies.

**Electroclinical syndromes are classified by the International League Against Epilepsy (ILAE) according to age [****4****]. In infancy, up to 40 % of epilepsies can be attributed to these epileptic encephalopathies, with West syndrome being the most common, accounting for approximately one third of cases [****5****], [****6****]. Figure** **3** **shows an overview of the genotype–phenotype correlation of the early epileptic encephalopathies.**	
Neonatal period	Early myoclonic encephalopathy and Ohtahara syndrome (EIEE)

	In the first three months of life, two severe epileptic encephalopathies manifest with neonatal convulsions and a suppression burst pattern on EEG: early myoclonic encephalopathy (EME) and Ohtahara syndrome. In EME, myoclonia are the predominant seizure type, which can be epileptic and nonepileptic. In Ohtahara syndrome, tonic spasms and prolonged tonic seizures are predominant. In EME, individual genetic and also metabolic causes are described, including glycine encephalopathy. For Ohtahara syndrome (early infantile epileptic encephalopathy [EIEE]), a number of different genetic causes have been found in recent years, including genes for a neuronal voltage-sensitive potassium channel (*KCNQ2*), a sodium channel of excitatory neurons (*SCN2A*), and a synaptic protein involved in the exocytosis of neurotransmitters (*STXBP1*), among numerous other rare genes [7]. Genotype–phenotype correlation is limited. The prognosis for neonatal epileptic encephalopathies is severe with relevant lethality, intellectual disability, and frequent transition to other more severe epilepsy syndromes, such as West syndrome or Lennox–Gastaut syndrome. Patients with mutations in *KCNQ2* appear to have a slightly more favorable prognosis with respect to epilepsy and, in some cases, better development with achievement of motor skills and simple communication [7], [8].
Infancy	Epilepsy of infancy with migrating partial seizures

	Malignant migratory epilepsy of infancy (EIMFS, incidence <1:100,000) is a very rare epileptic encephalopathy that manifests during the first weeks of life (usually around 7 weeks of life) with single focal motor seizures, progressing to a “storm phase” with many (up to several hundred) focal motor and subtle seizures daily and the typical clinical and EEG picture with “migrating,” status-like focal seizure patterns. Despite a later improvement in seizure frequency, the children develop a severe global developmental disorder [9]. To date, there is no clear superior treatment. A specific therapeutic approach of channel blockade with quinidine (with relevant risk of adverse drug reactions) eventually seems inferior to other classic antiepileptic drugs [10]. EIMFS has a high genotype–phenotype correlation, and mutations in the sodium-activated potassium channel (slack channel) encoded by *KCNT1* are predominant [11], [12].

	West syndrome (infantile spasms)

	West syndrome (infantile spasms) is the most common and prototypical epileptic encephalopathy. It is relatively common with an incidence of approximately 1:2,400 [6]. Infantile or epileptic spasm is the characteristic seizure type of a short tonic bilateral flexion [13]. West syndrome begins at the age of 2–12 months (often ∼6 months) [14]. There is usually a combination of a series of epileptic spasms, “hypsarrhythmia” on EEG, and developmental regression. Genetic causes can now be identified in a large proportion of children, showing a very high heterogeneity of genotype with only a few genes clustered, most notably *CDKL5*, *SCN2A*, *STXBP1*, *DNM1*, *KCNB1*, and also *SPTAN1* [9], [15], [16], [17].
	Dravet syndrome (SMEI)

	Dravet syndrome, with an incidence of approximately 1:22,000 [18], was first described by Charlotte Dravet as severe myoclonic epilepsy of infancy. Infants with prior normal development develop febrile, unilateral, or generalized clonic or tonic-clonic seizures, some as hemiclonic status epilepticus, at the age of 3–12 months. The seizures are often triggered by fever, infections, vaccinations, hyperthermia, or photostimulation [19]. In contrast to other epileptic encephalopathies, the EEG is often unremarkable. The prognosis seems to be explained mainly by the genetic disorder, in terms of a developmental encephalopathy [19]. De novo *SCN1A* gene mutations are found in up to 80 % of cases, and much less frequently mutations in *PCDH19* (X-dominant), *GABRA1*, *STXBP1*, or *KCNA2* are found [1], [9], [18], [20].
Childhood	Myoclonic atonic epilepsy

	This generalized epilepsy syndrome occurs in previously healthy children between 1 and 5 years of age, with a slight male dominance. Myoclonic seizures, sometimes subtle with a subsequent atonic phase that often leads to falls, are the typical seizure type, along with other generalized seizure types. The seizures can be difficult to treat; most helpful are valproate, ethosuximide, and the ketogenic diet. Mental development is variable, and up to 40 % of children develop mild to severe mental retardation [21], [22]. Repeatedly, alterations have been described in the genes *SLC2A1*, *SLC6A1*, *SCN1A*, *CHD2*, and most recently in a number of genes encoding RNA-binding proteins such as *HNRNPU* and *SYNCRIP* [22], [23], [24].

	Epilepsy-aphasia spectrum (CSWS and Landau–Kleffner syndrome)

	The epilepsy-aphasia spectrum includes a group of focal epilepsies and is associated with a risk of associated language disorders. These include continuous spikes and waves during sleep (CSWS) epilepsy, pseudo-Lennox syndrome, and Landau–Kleffner syndrome. They have in common a marked sleep activation of focal epileptic potentials toward a continuous status picture with generalized 1.5–3-Hz spike-wave series [25]. While in CSWS clinical seizures often occur during sleep, in pseudo-Lennox syndrome, other *petit mal* seizures such as absences or atonic seizures are present. In Landau–Kleffner syndrome, loss of expressive language skills is prominent, and clinical seizures may be absent [9], [25]. Genetic causes are found in approximately 20 % of patients. A subunit of the NMDA glutamate receptor, encoded by the gene *GRIN2A*, is most frequently affected. In addition, mutations in a number of other genes, encoding synaptic proteins, proteins involved in neuronal growth, neuronal transporters, and ion channels, like *MECP2, SLC9A6, KCNQ2, CNKSR2*, and *KCNA2*, were described [25], [26]. CSWS can also occur in children with mutations in neuronal tubulin genes (tubulinopathies). Tubulins are important neuronal structural proteins and associated with various central nervous system anomalies and developmental disorders [27], [28].

	Lennox–Gastaut syndrome

	Lennox–Gastaut syndrome (LGS) is more often than other childhood epilepsy syndromes a consequence of severe structural central nervous system damage involving the cerebral cortex and deep gray matter. Tonic seizures are predominant. Although LGS is often used and diagnosed for inclusion in clinical trials, children with presumed LGS can have different electroclinical epilepsies and classification is partially inconsistent. So here, we focus on better-defined EEs only.

**Figure 1:**
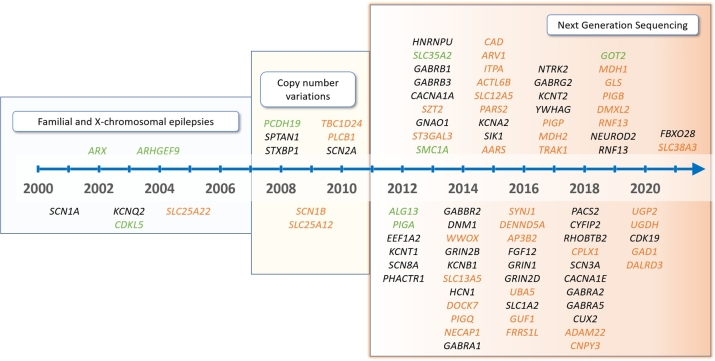
The current classification for monogenic developmental and epileptic encephalopathies (DEE, formerly EIEE; listed in the Online Mendelian Inheritance in Man database) comprises 102 genes, with the number doubling in the last 5 years. The genes are assigned in the timeline to the year of first description. In addition to this increasing list, many genes are not yet included and a recent study calculated that up to 1,000 more genes for developmental disorders can be identified [29]. Mode of inheritance: black: autosomal-dominant; red: autosomal-recessive; green: X-linked.

**The term DEE has now been included in the nomenclature of inherited disorders (Online Mendelian Inheritance in Man [OMIM]) for these genetic disorders with co-occurring epileptic seizures and abnormal EEG activity.** This reclassification allows a distinction of these monogenic etiology-specific syndromes from the electroclinical epilepsy syndromes with often diverse etiologic background. At the same time, the list of monogenic alterations as causes of these severe developmental disorders continues to grow and currently includes 102 different monogenic disorders (Figure 1). Table 1 lists recurrent examples of monogenic DEE. Derived from statistical calculations on genetic NDD and taking into account the genetic variation in large cohorts, it is assumed that another 1,000 genes for developmental disorders are still to be identified, which suggests a further increasing number of “epilepsy genes” [29]. Approximately one third of all epilepsies in infancy are pharmacorefractory, and within this group of pharmacorefractory epilepsies, a genetic causative alteration is found in over half of the children (53 %) [5]. Genetic epilepsies with onset before 3 years of age are often difficult to treat and classical antiseizure medications do not lead to sustained seizure freedom [1], [5]. Analogous to other disease groups, such as cancers, tumors, and cystic fibrosis, it is obviously desired to incorporate the knowledge of the specific genetic background into the choice of treatment [30].

Depending on the center and studies, probable causative genetic alterations are found in up to 50 % of children with early epilepsies, mostly in the form of monogenic disorders [31]. For the different age-related epileptic encephalopathies, the diagnostic yield differs, with a significantly higher yield after the introduction of high-throughput genetic sequencing (panel, exome) in recent years (Figure 2). Genetic testing is superior to the clinically suspected diagnosis, and a correct genetic cause can be predicted in only about 15 % of cases, limited to cases with good genotype–phenotype correlation, such as *PRRT2*-associated benign infantile epilepsy or *SCN1A*-associated Dravet syndrome [32], [33], [34].

**Table 1:** Exemplary list of recurrent and common monogenic developmental and epileptic encephalopathies (DEE).

Gene	Protein	OMIM phenotype	Functional consequence	Electroclinical syndrome	EEG	Development	Other phenotypes	Reference
**Voltage-gated neuronal sodium channels**								
***SCN1A***	Sodium channel, neuronal Type 1, alpha subunit, Na_V_1.1	Dravet (DEE6a) DEE6b	Loss of function, haploinsufficiency	Dravet syndrome	Normal at onset, later generalized spikes and abnormal background	Ataxia, moderate to severe intellectual disability	Epilepsy, generalized with febrile seizures plus 2; febrile seizures, familial 3A; migraine, familial hemiplegic 3	[35]
***SCN2A***	Sodium channel, neuronal Type 2, alpha subunit, Na_V_1.2	DEE11	Loss of function	Unspecified infantile encephalopathy with generalized seizures (age at onset >3 months), West syndrome, Lennox–Gastaut syndrome	Generalized spikes, hypsarrhythmia	Severe and global developmental disorder, autism	DEE/autism spectrum disorder, Seizures, benign familial infantile 3	[36]

			Gain of function	Ohtahara syndrome, neonatal infantile EE, epilepsy with migrating seizures in infancy	Burst suppression or multifocal spikes	Severe and global developmental disorder, associated movement disorder		
***SCN8A***	Sodium channel, neuronal Type 8, alpha subunit, Na_V_1.6	DEE 13	Gain of function	Infantile encephalopathy with focal seizures	Normal at onset, later multifocal spikes	Severe and global developmental disorder, early death	Benign infantile epilepsy 5, intellectual disability with/without ataxia	[37]

			Loss of function	Intellectual disability, autism	Multifocal and multiple spikes	Intellectual disability, autism spectrum disorder		
**Neuronal potassium channels**								
***KCNQ2***	Potassium channel, voltage-gated, subfamily Q, member 2, K_V_7.2	DEE 7	Loss of function (dominant-negative)	Ohtahara syndrome, neonatal EE	Burst suppression, or multifocal spikes	Severe and global developmental disorder, early death	Benign familial neonatal seizures (haploinsufficiency)	[38]

			Gain of function, increased potassium currents of voltage-gated potassium channels	Early myoclonic encephalopathy (EME), West syndrome	Burst suppression, hypsarrhythmia	Severe and global developmental disorder, early death		[39]
***KCNT1***	Potassium channel, subfamily T, Member 1, slack	DEE 14	Gain of function, increased potassium currents of sodium-activated potassium channels	Epilepsy with migrating seizures in infancy (EIMFS)	Migrating focal seizure patterns	Severe and global developmental disorder, early death	Autosomal dominant frontal lobe epilepsy 5	[40]
**Proteins with complex cellular functions, synaptic functions, and functions in neuronal dendritic growth**								
***STXBP1***	Syntaxin-binding protein 1, MUNC18-1	DEE 4	Loss of function, dysfunction of synaptic vesicle fusion (SNAP-SNARE complex)	Ohtahara syndrome, West syndrome, unspecified EE	Burst suppression, hypsarrhythmia, multifocal spikes	Severe intellectual disability, ataxia, associated movement disorders	Autism-tremor-ataxia	[41]
***CDKL5***	Cyclin-dependent kinase-like 5	DEE 2	Loss of function, reduced synaptic density and postsynaptic excitatory potentials	Rett-like syndrome with early seizures (Hanefeld variant), West syndrome, Lennox–Gastaut syndrome	Hypsarrhythmia, multifocal spikes, abnormal background	Severe and global developmental disorder, stereotypies, sleeping disorder		[42]

**Figure 2:**
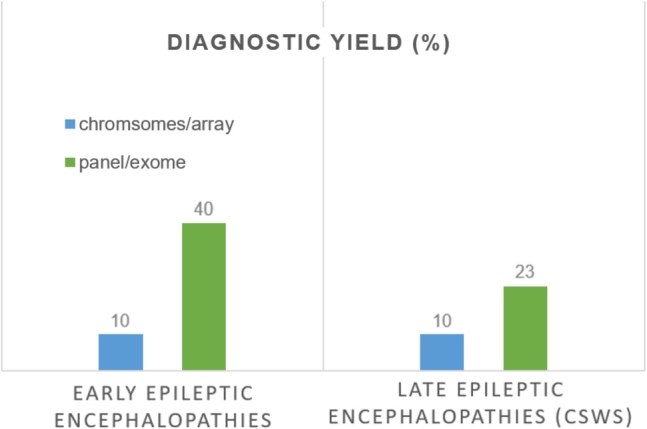
Diagnostic yield from different molecular genetic techniques. Chromosomal analyses and array CGH analyses have been conducted to detect causative genetic alterations in up to 10 % (e. g., microdeletions: del2211.2, Angelman syndrome, del1p36). With new exome-wide genetic analyses (next-generation sequencing), causative monogenic etiologies were identified in up to 40 % of children with early epileptic encephalopathies (West syndrome, epilepsy with migrating seizures of infancy, Dravet syndrome) and in more than 20 % of late epileptic encephalopathies (e. g., continuous spikes and waves during sleep [CSWS]). Modified after [25], [30], [43].

In our own cohort of more than 350 unselected children with epilepsy, screened between 2016 and 2022 with exome-wide sequencing techniques (panel, single exome, trio exome), around 50 % of children were found to harbor genetic alterations that are likely to contribute to their epilepsy syndrome. In children with likely pathogenic or pathogenic variants, these variants were found in more than 80 different disease-associated genes. Most single gene alterations were found in isolated cases and there were less than 25 genes that were affected in multiple children. This is in line with published studies on genetic causes of childhood epilepsy [5], [15], [33]. For more than 35 genes with a known cellular function, the identification of distinct variants had an impact on future counseling and treatment decisions, which included precision medicine approaches and also decisions for palliative care [5]. Recent publications have also described implications for treatment decisions, for epilepsy surgery, and for the relevance and care in elderly people with epilepsy [44], [45], [46].

Knowledge of the genetic and cellular causes will provide novel opportunities for individualized “precision therapy” of epilepsy and developmental disorders, and there is hope that the prognosis of these disorders will improve in the future. Following the increasing number of genetically identified causes of epilepsy, the number of publications on precision therapy has been increasing in recent years (Figure 3).

Precision therapy can be summarized as very different concepts.

**Table:** 

Specific therapeutic approaches for monogenic epilepsy syndromes (precision therapies)
**Empirical therapy of known neurological disorders**
Carbonic anhydrase inhibitors to reduce episodic ataxias and seizures in epilepsy-associated *CACNA1A* and *KCNA1* and presumably also in *KCNA2* [20], [47]
Avoidance of sodium channel blockers in *SCN1A*-associated Dravet syndrome [48].
Sodium channel blockers as the first therapeutic option in *KCNQ2*-associated neonatal seizures [49]
Use of sodium channel blockers and avoidance of levetiracetame in *PRRT2*-related infantile epilepsy [50]
**Direct antagonization of genetic effects**
Sodium channel blockers for gain-of-function mutations in *SCN2A* [51]

**Table:** 

Retigabine (currently not available) as a K_V_7/KCNQ-type potassium channel opener in *KCNQ2* encephalopathy [52]
Potassium channel blockers (aminopyridines) for gain-of-function mutations in *KCNA2* [20]
Primidone as a specific channel blocker in *TRPM3*-associated epilepsy (Transient Receptor Potential Melastatin 3) [53], [54]
**Use of agonists in cases of reduced ion channel or receptor function**
L-serine therapy for *GRIN2B*- and other GRIN-associated developmental disorders [55], [56]
**Inhibition of overactive cellular signaling pathways**
mTOR inhibitors in tuberous sclerosis [57]
Alpelisib in *PIK3CA*-associated overgrowth syndrome [58]
**Correction of disturbed protein structure**
Chemical chaperones can improve various aspects of impaired protein function in missense mutations. In animal models, this has been shown for sodium and phenyl butyrate in LGI1-associated epilepsy and in *STXBP1*-associated encephalopathy [59], [60]. US study: NCT04937062, contact the author for individual treatment protocols.
**Protein replacement/enzyme replacement therapy**
Intrathecal enzyme replacement therapy for neuronal ceroid lipofuscinosis [61]
**Gene therapy (ASO, CRISPR/Cas9, gene replacement therapy)**
Several novel concepts are on the horizon and await first-in-man studies. These include antisense oligonucleotides (ASOs) to reduce the expression of overactive ion channels or proteins as a specific therapeutic approach for numerous epilepsies due to mutations with a dominant-negative effect. The absence of an allele in these disorders (haploinsufficiency) is usually associated with a less severe phenotype than mutations that additionally disrupt the healthy allele. In these disorders and epilepsies, carriers of whole-gene deletions are less severely affected than carriers of single point mutations (e. g., in *SCN2A*, *KCNT1*, *KCNA2*, *SPTAN1*). ASOs have already been successfully tested in animal models and first-in-man trials are underway for various monogenic epilepsies, similar to the successful implementation of ASOs in spinal muscular atrophy [62]. Another ASO technology has also been introduced to increase protein levels in disorders with a loss-of-function mechanism from haploinsufficiency. Targeted Augmentation of Nuclear Gene Output (TANGO) has been successfully used in mouse models of Dravet syndrome [63].

As the number of treated children with rare monogenic epilepsies is increasing, so is the knowledge of the benefit of specific therapeutic approaches. Some early gene-based “precision therapies,” such as quinidine for *KCNT1*-associated epilepsy with migrating seizures in infancy, have now failed to confirm high expectations in larger patient groups, and common antiseizure medications have been shown to be superior to precision therapy approaches [10]. So far genetic findings will have a positive impact on therapy in isolated cases only and it is important to counsel parents and relatives on the limits of precision medicine, as genetic diagnosis can give too much hope and about one fifth of parents expect immediate improvements in therapy [64].

The concept of epileptic encephalopathy in the sense that epilepsy determines developmental prognosis according to the type, duration, and disease onset of the syndrome and timing of treatment is increasingly being challenged. Recent epidemiological studies show that etiology is the most important risk factor for developmental disability [5]. At the same time, the EPISTOP study demonstrated that nonspecific, prophylactic antiepileptic therapy with vigabatrin reduces the risk of epilepsy and seizures in children with tuberous sclerosis. Unfortunately, however, intellectual disability and especially autism at 2 years of age occurred independently and were not significantly improved by preventive anti-epileptic treatment [65]. These findings question a direct connection between epilepsy, EEG activity, and a subsequent developmental disorder and highlight the need to develop new treatment approaches with disease-modifying capacities. In a mouse model, neurocognitive and behavioral deficits were rescued by targeting the overactive mTOR signaling pathway. In Germany, we currently lead a study of preventive therapy with sirolimus, an mTOR inhibitor, aiming to randomize newborns and young infants with tuberous sclerosis throughout Germany in order to reduce the 40–50 % risk of mental retardation in affected persons.

**Figure 3:**
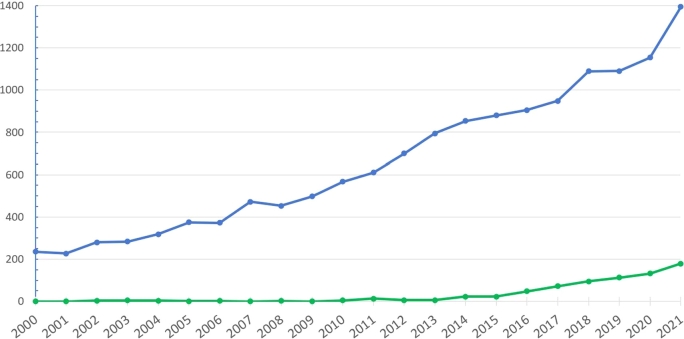
Number of publications in PubMed for search terms “epilepsy and gene” (blue) and “precision medicine and epilepsy” (green). The number of publications on precision medicine (green) has rapidly increased during the last 5 years, following the increase in identified genetic causes (blue) (source: www.pubmed.gov). Modified after [30].

**Figure 4:**
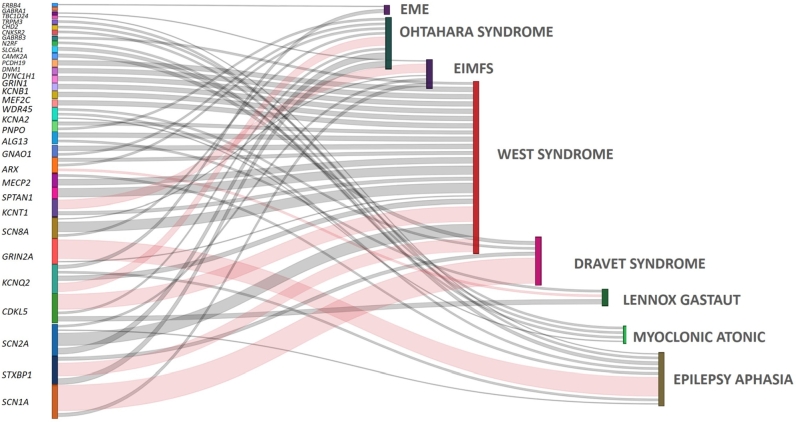
Genotype–phenotype correlation of genes associated with developmental and epileptic encephalopathies (DEE) and age-related electroclinical epileptic encephalopathies (EE), personally curated by the author from the Online Mendelian Inheritance in Man database, reviews and genotype–phenotype studies, not scaled (neonatal: early myoclonic encephalopathy [EME], Ohtahara syndrome; infantile: epilepsy with migrating seizures in infancy [EIMFS], West syndrome, Dravet syndrome; childhood: Lennox–Gastaut syndrome, myoclonic-atonic epilepsy, epilepsy-aphasia spectrum [continuous spikes and waves during sleep, Landau–Kleffner syndrome]). Single encephalopathies such as EIMFS (*KCNT1*) or Dravet syndrome (*SCN1A*) have a high genotype–phenotype correlation (red lines). By contrast, West syndrome has a low genotype–phenotype correlation and a high number of different genetic alterations have been identified [1], [7], [9], [15], [30], [33], [43].

Monogenic DEE are rare diseases and the respective phenotypic variation is only slowly being recognized. The prognosis of childhood epileptic and genetic encephalopathies remains poor despite advances in diagnosis and therapy. Knowledge of genotype–phenotype correlations between single genes and the respective age-related epileptic encephalopathy is important to better understand specific cellular mechanisms leading to distinct epilepsy syndromes (Figure 4). Natural history studies and the implementation of patient registries through collaborations with parental and patient advocacy groups (such as https://stxbp1-ev.de/ or https://www.cdkl5-verein.de/) are important to define patient-centered outcomes and to provide access to new therapies.

## Conclusion for practice

– Genetic causes are increasingly recognized and cellular mechanisms in childhood epilepsies are better understood.
– Because of the limited genotype–phenotype correlation and the increasing number of monogenic disorders, primary whole-exome sequencing should be performed.
– By studying the pathophysiological processes, we hope to improve prognosis of these severe childhood developmental disorders in the long run.
